# Decoding Flavonoid Metabolism for Nutritional Enhancement: A Transcriptome–Metabolome Integration Study of Biosynthesis in Edible *Chrysanthemum indicum* L.

**DOI:** 10.3390/foods14111896

**Published:** 2025-05-26

**Authors:** Chengxiang Wang, Yong Su, Min Wei, Qiaosheng Guo, Qingjun Zou, Tao Wang

**Affiliations:** 1 Institute of Chinese Medicinal Materials, Nanjing Agricultural University, Nanjing 210095, China; 2023104141@stu.njau.edu.cn (C.W.); 18119845526@163.com (Y.S.);; 2 China Resources Sanjiu Medical & Pharmaceutical Co., Ltd., Shenzhen 518110, China

**Keywords:** *Chrysanthemum indicum* L., flavonoids, transcriptome, metabolome

## Abstract

*Chrysanthemum indicum* L. is characterized by a high concentration of flavonoid compounds, which exert multifaceted influences on the organoleptic properties, chromatic stability, and therapeutic efficacy of capitulum-derived extracts. These components exhibit diverse biological activities, including heat-clearing, antibacterial, and hepatoprotective properties. A novel white *C. indicum* variant lacking linarin was recently identified, but its metabolic and transcriptional differences from traditional yellow varieties remain unclear. This study compared flavonoid metabolism in white mutant (BHYJ) and yellow (HJ06) varieties through integrated metabolomic and transcriptomic analyses. Metabolomics identified 491 flavonoids, revealing distinct accumulation patterns: BHYJ accumulated dihydroflavones/chalcones (eriodictyol, hesperetin-8-*C*-glucoside-3′-*O*-glucoside, naringenin chalcone), while HJ06 showed higher flavones/flavonols (linarin, rhoiflolin, vitexin, rutin, nicotiflorin). Transcriptomics identified 43 differentially expressed enzyme genes, with key regulators *FNSII*, *F3′H*, and *F3H* showing expression patterns correlating with metabolite profiles. Integrated analysis revealed metabolic divergence at the naringenin node: BHYJ produced less naringenin than HJ06 and preferentially channeled it toward eriodictyol synthesis rather than linarin production. This metabolic shift explains the reduced linarin accumulation in BHYJ. Experimental validation confirmed the coordinated expression patterns of key enzymes. These findings provide foundational insights into transcriptional regulation of flavonoid divergence in pigmented *C. indicum* varieties, establishing a framework for elucidating enzymatic control of flavonoid biosynthesis in capitulum development.

## 1. Introduction

*Chrysanthemum indicum* L., a perennial herbaceous plant belonging to the Asteraceae family and the genus Chrysanthemum in China, is recognized for its dual use as both a medicinal herb and a food source [1]. Currently, it is listed by the Ministry of Health as a Chinese herb approved for use in health foods, with 21 domestic health food products containing *C. indicum* documented in the Health Food Prescription Database [2]. The dried capitulum of *C. indicum* (medicinally referred to as “Flos Chrysanthemi Indici”) is renowned for its heat-clearing, detoxifying, liver-calming, and fire-draining properties [3]; as a key raw material in 999 Cold and Flu Granules, it commands substantial annual demand. In addition to its medicinal applications, *C. indicum* possesses multiple values, including edible, ornamental, and chemical raw material uses [4]. For instance, *C. indicum* tea is a widely consumed traditional beverage and is frequently incorporated into herbal tea blends in southern China. The food value of *C. indicum* extends beyond traditional uses to modern food processing, where it serves as a natural food additive in meat and dairy products, functioning as a preservative and sterilizer [5]. Additionally, it is utilized as a natural coloring and flavoring agent in food products [6]. The primary active components in *C. indicum* encompass flavonoids, terpenoids, organic acids, and polysaccharides, with flavonoid compounds being the primary focus of research [7]. According to the Chinese Pharmacopoeia [8], linarin is established as the quality control marker for *C. indicum.* Although *C. indicum* is classified as a polyphyletic species in the Flora of China [3], research on its diversity has predominantly centered on morphological traits, including leaf shape, leaf arrangement, corymb structure, and stem–leaf indumentum, with limited investigation into its color characteristics. In ornamental horticulture, researchers frequently investigate the underlying causes of flower color variation and explore their applications in breeding programs. Techniques such as quantitative analysis of chrysanthemum flower color phenotypes, petal structure examination, and flower pigment composition analysis are widely used to elucidate the mechanisms of flower color formation and to establish a theoretical basis for chrysanthemum flower color breeding [9]. In contrast, within the field of medicinal plants, the emphasis shifts to investigating the influence of flower color variation on the active constituents and medicinal efficacy of floral herbs. For example, yellow and white medicinal *Chrysanthemum morifolium* Ramat exhibit distinct therapeutic properties: white *C. morifolium* is renowned for its heat-clearing effects, while yellow *C. morifolium* is valued for its liver-calming properties. Variations in flower color can induce changes in the metabolic pathways of medicinal plants, leading to fluctuations in the concentrations of active constituents and potentially affecting the stability of medicinal resources [10]. Li et al. employed advanced techniques, including colorimeters and ultra-performance liquid chromatography (UPLC), to assess the color of various *C. indicum* samples, with the goal of performing correlation analyses to rapidly predict the intrinsic quality of Chrysanthemi Indici Flos (traditional Chinese medicine) [11]. Recently, a white bud mutant was found in the HJ06 population, and the fact that it has no detectable linarin component makes it an ideal material for studying the relationship between flower color change and composition as well as exploring the mechanism of flavonoid synthesis. Although significant color variation was observed exclusively in ray flowers, it remained uncertain whether disk flowers exhibited similar variation. Therefore, this study segregated the experimental materials into ray and disk flowers, which were analyzed separately.

Flavonoid compounds, which are derived from the flavone (2-phenylchromone) core structure, constitute essential medicinal components in *C. indicum* [12]. These compounds are typically present in plant tissues as glycosides conjugated with sugars or as carbon glycosides [13]. As secondary metabolites, flavonoids display significant biological activity and are extensively distributed within plants, regulating diverse functions including growth hormone modulation, cell wall development, root growth control, pollen attraction, UV protection, and defense against pests and diseases [14]. For *C. indicum* processed foods, flavonoids are essential for flavor, color, and gloss formation [15]. The flavonoids in *Chrysanthemum indicum* L. exhibit a range of healthcare functions, primarily including antioxidant [16], antibacterial [17], antitumor [18], cardiovascular protective [19], and liver functions [20]. The flavonoid biosynthesis pathway diverges from phenylpropanoid biosynthesis. Initially, chalcone is synthesized via the condensation of coumaryl-CoA and malonyl-CoA, catalyzed by chalcone synthase (*CHS*) [21]. The second step in flavonoid biosynthesis involves chalcone isomerase (*CHI*), which catalyzes the stereospecific cyclization of chalcone to produce naringenin, a key intermediate in the flavonoid pathway [22]. Flavonoid compounds, including linarin, luteolin, acacetin, apigenin, luteolin-7-*O*-glucoside, and kaempferol, have been isolated from the capitulum of *C. indicum* [23]. Since the late 20th century, research on flavonoids has significantly expanded in the fields of genetic and metabolic engineering [24]. Optimizing the metabolic pathways of *C. indicum* can provide valuable insights for its cultivation and breeding.

The integration of transcriptomic and metabolomic analyses serves as a robust strategy for elucidating physiological mechanisms and identifying key genes. In recent years, integrated transcriptomic and metabolomic analyses have been increasingly employed to study plant secondary metabolites and their regulatory pathways. Jiang et al. utilized this approach to elucidate the metabolic pathways of terpenoids and flavonoids in different parts of *C. indicum*, revealing key regulatory factors [4]. Furthermore, Liao identified three *7-O-UGT* genes within two correlation networks that are crucial for the biosynthesis of flavonoid-7-*O*-glucoside in *C. indicum* [25]. BHYJ and HJ06 differed significantly in flower color and linarin composition, and it is hypothesized that the linarin metabolic pathway may be different in different-colored *C. indicum*, with differences in related enzymes and compounds. Through comprehensive metabolomic and transcriptomic analyses, researchers further investigated the synthesis mechanisms of flavonoid components in different *C. indicum* varieties. This study identified and preliminarily validated key genes involved in these processes, aiming to establish a foundation for evaluating the quality of *C. indicum* capitula, guiding the rational utilization of its medicinal properties, and providing novel insights for improving flower quality and developing new varieties through molecular breeding and metabolic engineering techniques. These findings lay the groundwork for the development of *C. indicum* with enhanced flavor and therapeutic efficacy.

## 2. Materials and Methods

### 2.1. Materials

**Figure 1:**
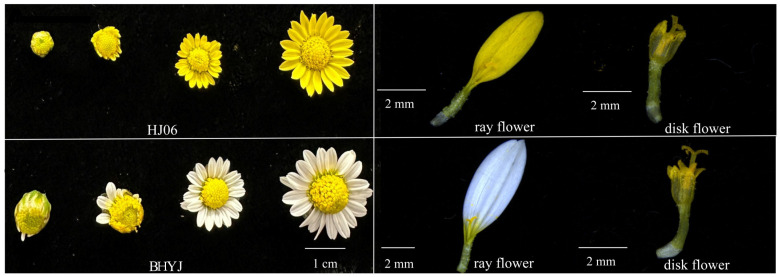
Comparison of the shapes of HJ06 and BHYJ.

The traditional *C. indicum* (with yellow ray flowers) and the white ray-flowered *C. indicum* used in this experiment were provided by China Resources Sanjiu Medical & Pharmaceutical Co., Ltd. (Shenzhen, China). Both varieties were collected from Yangxin County, Huangshi City, Hubei Province, China, and identified by Associate Prof. Wang Tao of Nanjing Agricultural University as *C. indicum* of the genus Chrysanthemum (family Asteraceae). In this study, we randomly selected three sampling points in the *C. indicum* experimental field for sampling, and the sampling criterion was the same growth and the same external morphological characteristics of *C. indicum* (a ray flower blooming rate of 70% and a disk flower blooming rate of 50%). Samples were mixed and divided into triplicates at the end of sampling as three biological replicates, rapidly frozen in liquid nitrogen, and stored at −80 °C. The typical capitulum of *C. indicum* was coded as HJ06, and the white capitulum was coded as BHYJ. The disk flowers of HJ06 were labeled as Hg, and the ray flowers as Hs. Similarly, the disk flowers of the white *C. indicum* were labeled as Bg, and the ray flowers as Bs (Figure 1).

### 2.2. Detection of Linarin

Approximately 0.25 g of the powdered sample (passed through a third sieve) was accurately weighed and placed in a stoppered conical flask. Then, 100 mL of 100% methanol was precisely added, and the mixture was subjected to heating reflux for 3 h. The resulting filtrate was filtered through a 0.22 µm microporous organic membrane and used for ultra-performance liquid chromatography (UPLC) analysis. The mobile phase consisted of acetonitrile (solvent A) and a 0.1% phosphoric acid solution (solvent B). The gradient elution program was set as follows: 0 min, 0% A/100% B; 3 min, 0% A/100% B; 5 min, 25% A/75% B; 8 min, 45% A/55% B; 15.5 min, 27% A/73% B; and 18.5 min, 0% A/100% B [8].

### 2.3. Electronic Tongue Analysis

Each sample of (2.00 ± 0.05 g) steamed and dried *C. indicum* (BHYJ and HJ06) was weighed and brewed in 150 mL of boiling water for 30 min, and then the supernatant was collected by centrifugation and filtered through a 0.45 μm filtration membrane, and the filtrate was kept for testing. At the same time, three auxiliary solutions were prepared: a reference solution (2.2365 g of potassium chloride and 0.045 g of L-tartaric acid were mixed with 1 L of pure water), a positive electrode cleaning solution (500 mL of pure water mixed with 300 mL of anhydrous ethanol and then added to 8.3 mL of hydrochloric acid to 1 L), and a negative electrode cleaning solution (7.46 g of potassium chloride, 500 mL of pure water, 300 mL of anhydrous ethanol, and 0.56 g of potassium hydroxide were mixed and fixed to 1 L; the solution was mixed and fixed to 0.56 g of potassium hydroxide; potassium hydroxide was mixed and fixed to 1 L). The test was carried out using the electronic tongue model SA-402B from Insent, Japan, whose sensor array consists of a positive electrode (C00, AE1, and reference electrode) and a negative electrode (CT0, CA0, AAE, and reference electrode). Before testing, each solution was poured into a special beaker and the sensor was cleaned sequentially with positive and negative cleaning solutions for 90 s, followed by equilibration in the reference solution for 120 s, and the process was repeated once. When the sample is tested, the sensor collects data for 30 s and then quickly cleans twice and returns to the reference solution to determine the residual flavor value. The two sensory characteristics (bitterness, astringency) of each sample were evaluated by detecting changes in membrane potential caused by electrostatic interactions or hydrophobic interactions between various flavor substances and artificial lipid membranes [15]. Three technical replicates were set up for each sample, and each replicate measurement was taken four times in an experimental environment of 25 ± 0.5 °C and 70 ± 2% RH. The data were analyzed by rounding off the 1st measurement value and taking the 30th second intensity value of the last three times, which were combined with the R package (version 4.4.3) to perform PCA and taste radargram analysis.

### 2.4. Extraction of Flavonoids

The samples were processed by vacuum freeze-drying (Scientz-100F lyophilizer), and then ground into powder using a grinder (MM 400, Retsch) at 30 Hz for 90 s. Subsequently, 50 mg of the powder sample was weighed accurately by an electronic balance (MS105DM), and then added to 1200 μL of pre-cooled 70% methanol aqueous internal standard extracts at −20 °C, and then subjected to vortexing six times (30 s each, with intervals of 30 min). After six vortex treatments (30 min and 30 s each), the sample was centrifuged at 12,000 rpm for 3 min, and the final supernatant was filtered through a 0.22 μm microporous membrane and stored in the injection bottle for UPLC-MS/MS analysis.

### 2.5. Instrument Parameter Settings

The data acquisition system consisted of an ultra-performance liquid chromatography (UPLC) system coupled with Tandem Mass Spectrometry (MS/MS) using an Applied Biosystems 6500 QTRAP instrument. The liquid chromatography conditions were as follows: column, Agilent SB-C18 (1.8 µm, 2.1 mm × 100 mm); mobile phase A, ultrapure water with 0.1% formic acid; mobile phase B, acetonitrile with 0.1% formic acid; gradient elution program, 5% B to 95% B over 9.00 min, held at 95% B for 1 min, returned to 5% B by 11.10 min, and equilibrated at 5% B until 14 min; flow rate, 0.35 mL·min^−1^; column temperature, 40 °C; injection volume, 2 μL. The mass spectrometry conditions included triple quadrupole (QQQ) scans performed on the AB6500 QTRAP UPLC/MS/MS system equipped with an ESI Turbo Ion Spray interface. The system was operated in positive and negative ion modes using Analyst 1.6.3 software. The ESI source parameters were as follows: ion source, turbo spray; source temperature, 550 °C; ion spray voltage (IS), 5500 V (positive ion mode) and −4500 V (negative ion mode). The gas pressures were set as follows: Gas I, 50 psi; Gas II, 60 psi; and Curtain Gas (CUR), 25 psi. Collision-induced ionization was set to a high level. QQQ tuning and mass calibration were performed using polyethylene glycol solutions at concentrations of 10 and 100 μmol·L^−1^, respectively. MRM scans in QQQ mode used nitrogen as the collision gas, with optimized declustering potential (DP) and collision energy (CE) settings for each MRM ion pair. Specific MRM ion pairs, including the parent ion (Q1) and fragment ion (Q3), were monitored during each elution period corresponding to the eluted metabolites.

### 2.6. Metabolomics Data Processing

Metabolite identification is based on metabolite accurate mass, a secondary mass spectrometry (MS2) fragment, MS2 fragment isotope distribution, and retention time (RT). Through the intelligent secondary spectrum matching method, the secondary spectrum and RT of metabolites in the project samples are matched with the secondary spectrum and RT of the MWDB (metware database) one by one. The MS tolerance and MS2 tolerance are set at 20 ppm, and the RT tolerance is 0.2 min. Based on the above information of the samples and the matching score of the compounds in the database, the compounds’ identification level was determined, in which Level1 had the highest accuracy, and the order of accuracy of Level1 to Level3 was decreasing. Metabolite quantification was accomplished using the multiple reaction monitoring (MRM) mode of triple quadrupole mass spectrometry, in which the quadrupole first screens the precursor ions (parent ions) of the target compounds and excludes ions corresponding to other molecular weight compounds in order to exclude interferences initially, and then the precursor ions are induced to ionize by the collision chamber and then break to form many fragment ions. The precursor ions are fractured after induced ionization in the collision chamber to form many fragment ions, and the fragment ions are then filtered through the triple four-stage rod to select a desired characteristic fragment ion, which excludes the interference of non-target ions, and results in a more accurate and reproducible quantification [26]. After obtaining the metabolite profiling data of different samples, the relative concentration of metabolites was calculated using the ratio of the amount of reference internal standard to the peak area of the internal standard, and peak area integration was performed on all the peaks of the compounds’ chromatograms, the integration was corrected for the mass spectrometry peaks of the same metabolite in different samples [27], and the data were displayed as the mean of three replicates plus or minus the standard error of the mean (mean ± SEM). Principal component analysis (PCA), volcano plot analysis, and correlation analysis were performed using the Metware Cloud platform (https://cloud.metware.cn/#/h-ome, accessed on 1 February 2025).

### 2.7. RNA Extraction and Transcriptome Sequencing

Total RNA was extracted from disk flowers (Bs/Hs) and ray flowers (Bg/Hg) of two distinct *Chrysanthemum indicum* varieties using the SteadyPure Plant RNA Extraction Kit (Aikerui Biotechnology Co., Ltd., Changsha, China). All samples were collected at the same developmental stage (70% of ray flowers open and 50% of disk flowers open), with three biological replicates per sample. Qualified RNA samples were submitted to Metware Biotechnology Co., Ltd. (Woburn, MA, USA) for cDNA library construction, followed by high-throughput sequencing on the Illumina HiSeq platform with paired-end 150 bp reads.

### 2.8. Transcriptome Data Processing

Ribosomal RNA (rRNA) was removed from total RNA to isolate mRNA, which was then fragmented using a fragmentation buffer to generate short RNA fragments. Subsequently, the short RNA fragments were used as templates to synthesize the first-strand cDNA using random hexamer primers. Next, the second-strand cDNA was synthesized using a reaction mixture containing buffer, dNTPs (dUTP, dATP, dGTP, and dCTP), and DNA polymerase I, followed by purification with AMPure XP beads. The purified double-stranded cDNA was subjected to end repair, A-tailing, and sequencing adapter ligation. Subsequently, fragment size selection was performed using AMPure XP beads, followed by PCR amplification to generate the final cDNA library. Bioinformatic analysis was performed to predict alternative splicing events, optimize gene structures, identify novel genes, and quantify gene expression levels based on read alignment results. Differentially expressed genes (DEGs) were identified across samples, followed by functional annotation and enrichment analysis [1]. DEGs were identified using DESeq2 with the following thresholds: |log_2_(fold change)| > 0 and adjusted *p*-value (padj) < 0.05. TGene expression levels were quantified using FPKM (fragments per kilobase of transcript per million mapped reads) values, which represent the relative expression levels of DEGs. Functional enrichment analysis, including Gene Ontology (GO) and Kyoto Encyclopedia of Genes and Genomes (KEGG) pathways, was performed on DEGs with a corrected *p*-value < 0.05.

### 2.9. Integrated Analysis of Metabolomics and Transcriptomics

The screening results of differentially expressed genes (DEGs) in the flavonoid biosynthesis pathway and associated metabolites were used to map transcriptomic and metabolomic data onto the KEGG pathway map. KEGG enrichment analysis was performed with a threshold of Pearson correlation coefficient ≥ 0.8 to investigate the correlation between genes and metabolites. Genes involved in the flavonoid biosynthesis pathway were identified through KEGG pathway annotation, specifically within pathways ko00940 (flavonoid biosynthesis), ko00941 (flavone and flavonol biosynthesis), ko00942 (anthocyanin biosynthesis), ko00943 (isoflavonoid biosynthesis), and ko00944 (flavonoid and flavonol biosynthesis). Weighted Gene Co-Expression Network Analysis (WGCNA) was used to analyze the co-expression patterns of genes across samples. WGCNA is based on high-throughput RNA-seq data and constructs gene co-expression networks to divide genes into modules, which are then analyzed for their associations with specific phenotypes or clinical traits [28]. A co-expression network was constructed, encompassing key regulatory genes and transcription factors (TFs) from highly correlated modules related to the flavonoid biosynthesis pathway. The regulatory interactions between these elements were visualized using Cytoscape software (version 3.10.1).

### 2.10. RT-qPCR Analysis

To validate the transcriptome sequencing results, a subset of the total RNA used in the RNA-Seq analysis was subjected to validation via reverse transcription quantitative PCR (RT-qPCR). The primary objective was to identify DEGs linked to flavonoid biosynthesis pathways, enabling spatial and temporal expression analysis. The RT-qPCR primers were designed using Primer 3 software (version 0.4.0; see Table S1). Total RNA was extracted using the SteadyPure Plant RNA Extraction Kit (Accurate Biology Co., Ltd., Changsha, China), followed by reverse transcription into cDNA using the Evo M-MLV Reverse Transcription Premix Kit (Accurate Biology Co., Ltd., Changsha, China). RT-qPCR assays were performed using the SYBR Green Pro Taq HS Premix qPCR Kit (Accurate Biology Co., Ltd., Changsha, China). The 20 μL amplification reaction mixture consisted of 1 μL cDNA, 0.4 μL each of forward and reverse primers, 10 μL of 2× SYBR Green Pro Taq HS Premix, and 8.2 μL of ultrapure water. The amplification protocol consisted of an initial denaturation step at 95 °C for 30 s, followed by 40 cycles of denaturation at 95 °C for 5 s, annealing at 55 °C for 30 s, and extension at 72 °C for 30 s. The relative gene expression levels were calculated using the 2-ΔΔCT method, with EF1α (forward primer: 5′-GGTCAGATTGGAAACGGTTAT-3′; reverse primer: 5′-AGGTGGGTATTCAGCAAAGG-3′) as the reference gene. Each experiment was performed in triplicate.

## 3. Results

### 3.1. Metabolomic Differential Analysis

Figure 1 presents a morphological comparison of *C. indicum* with varying flower colors, demonstrating pronounced color variations in the ray flowers of BHYJ and HJ06, whereas distinctions in the disk flowers are less pronounced. Based on these observations, this study classifies *C. indicum* flowers into two categories, ray and disk flowers, for further analysis. The UPLC analysis revealed that linarin was absent in BHYJ (Figure S1).

#### 3.1.1. Analysis of Flavonoid Metabolomes

**Figure 2:**
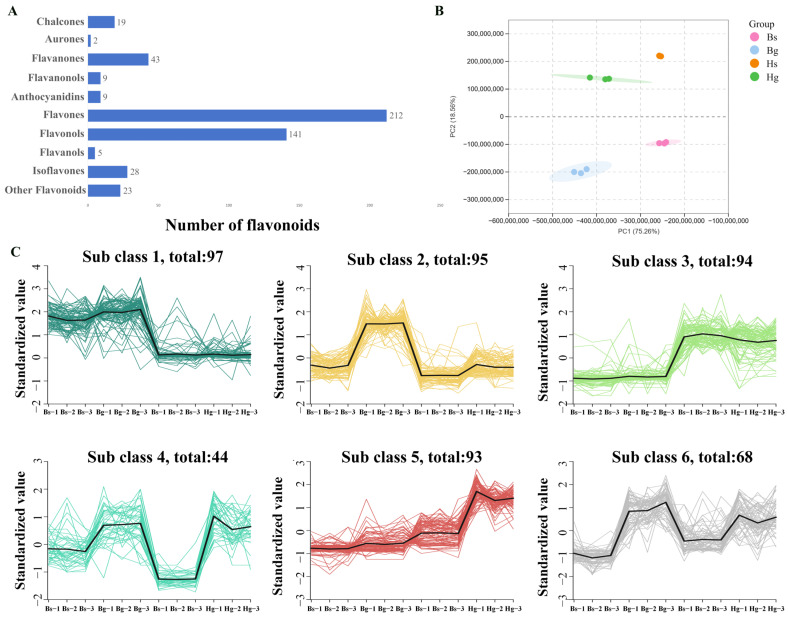
Analysis of the flavonoid metabolism spectrum. (**A**) The number of flavonoid metabolites; (**B**) PCA analysis (Hg: the disk flowers of HJ06; Hs: the ray flowers of HJ06; Bs: the disk flowers of BHYJ; Bg: the ray flowers of BHYJ); (**C**) K−means cluster analysis of the DAMs (The different subclusters are separated by different colors, sub class represents the cluster number of compounds with the same trend, and the number after total indicates the number of compounds in the cluster).

The UPLC-MS analysis characterized a total of 491 flavonoid compounds in the ray and disk flowers of HJ06 and BHYJ, including 28 isoflavones, 5 flavanols, 141 flavonols, 212 flavones, 9 anthocyanins, 9 dihydroflavonols, 43 dihydroflavones, and 21 chalcones (Figure 2A), MRM detection of multimodal maps is shown in Figure S2. Similar flavonoid compositions were observed in both the disk and ray flowers of HJ06 and BHYJ. To further investigate the metabolic distinctions and accumulation patterns of metabolites, principal component analysis (PCA) and cluster analysis were performed. PCA of flavonoid metabolites revealed distinct profiles in the ray and disk flowers of BHYJ and HJ06, with no overlapping regions in the projection, indicating significant differences in flavonoid accumulation patterns between the two varieties. The first principal component (PCA1) explained 28.82% of the variance, and the second principal component (PCA2) explained 44.21% of the variance (Figure 2B). K-means clustering of differentially accumulated metabolites (DAMs) was performed to analyze trends in the relative content of DAMs across different subgroups. Metabolite clustering in the nine plots revealed significant changes, as illustrated in Figure 2C. The first and third groups of metabolites exhibited opposing accumulation trends. We hypothesize that these groups best represent the flavonoid components associated with floral color variation, while the third group likely contains components responsible for the accumulation of linarin.

#### 3.1.2. Analysis of Flavonoid Differentially Accumulated Metabolites (DAMs)

**Figure 3:**
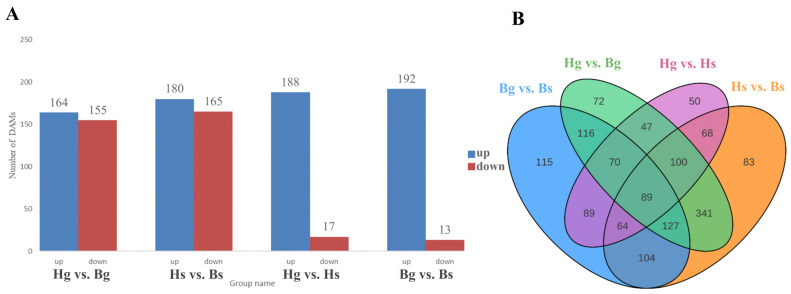
Differential metabolite analysis of flavonoids. (**A**) Differential accumulation diagram (Hg: the disk flowers of HJ06; Hs: the ray flowers of HJ06; Bs: the disk flowers of BHYJ; Bg: the ray flowers of BHYJ); (**B**) Venn diagram.

To investigate interspecies variations in flavonoid compounds between the disk and ray flowers of yellow and white *C. indicum*, we identified 319 differentially accumulated metabolites (DAMs) in the disk flowers of BHYJ and HJ06, and 345 DAMs in the ray flowers. Analysis of metabolite differences between the ray and disk flowers within the same *C. indicum* variety revealed 205 DAMs in HJ06 and 205 DAMs in BHYJ. Notably, higher levels of flavonoid compounds were observed in HJ06 compared to BHYJ (Figure 3A). Similarly, higher levels of flavonoid compounds were detected in the disk flowers than in the ray flowers. The Venn diagram analysis of DAMs revealed that Bg_vs_Bs had the highest number of DAMs, whereas Hg_vs_Hs had the lowest (Figure 3B).

#### 3.1.3. Cluster Analysis of Flavonoid Metabolites

**Figure 4:**
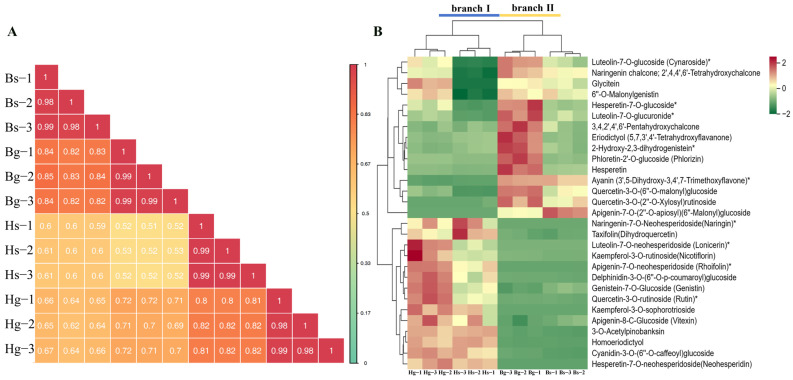
Comprehensive analysis of flavonoids. (**A**) Pearson correlation analysis of all sample metabolites; (**B**) 30 types of flavonoids identified in flavonoid biosynthesis pathways (* indicates the compounds’ identification level was Level).

To assess the reproducibility among samples, Pearson correlation analysis was conducted, revealing high reproducibility between samples (Figure 4A). Hierarchical clustering analysis of 491 flavonoid compounds revealed that Hg and Hs were grouped in branch I, while Bg and Bs were grouped in branch II. These flavonoid compounds were further classified into four clades—clade I (elevated levels in Hs), clade II (elevated levels in Hg and Hs), and clades III and IV (elevated levels in Bg and Bs)—as shown in Figure S3. Based on KEGG pathway annotation, 30 significantly changed metabolites (SCMs) were identified in pathways related to flavonoid biosynthesis, isoflavonoid biosynthesis, anthocyanin biosynthesis, and flavone and flavonol biosynthesis, including compound number, parent ion (Q1), product ion (Q3), chemical formula, CAS number, and ionization model, among others, through the use of a database and previous studies (Table 1). Cluster analysis of these 30 SCMs revealed that Hg and Hs were grouped in branch I, while Bg and Bs were grouped in branch II (Figure 4B). BHYJ and HJ06 showed significant differences in the four flavonoid-related biosynthetic pathways. Furthermore, the 30 SCMs were classified into two main clades: clade I and clade II. In clade I, compounds such as naringenin chalcone and eriodictyol showed higher levels in BHYJ compared to HJ06, whereas in clade II, metabolites such as naringenin and rutin were more abundant in HJ06. A preliminary hypothesis proposes that the flavonoid biosynthesis pathways in BHYJ and HJ06 diverge at the naringenin node.

#### 3.1.4. Electronic Tongue Analysis of BHYJ and HJ06

The bitterness of *C. indicum* is an important indicator reflecting the changes in its internal composition and efficacy value, and electronic tongue analysis of the boiled liquids of BHYJ and HJ06 was conducted to further investigate whether the differences in the flavonoid metabolites of the two types of *C. indicum* could cause the changes in their bitterness. The electronic tongue data of the four groups of samples were histogrammed to obtain Figure S4, from which the differences in the intensity of the same flavor among the four groups of samples can be more intuitively seen. Astringency was not detected in any of the four samples. In terms of bitterness, Bg had the strongest bitter taste and Hs had the weakest bitter taste. BHYJ had a lower bitterness than HJ06 for both disk and ray flowers, which may be related to the accumulation of flavonoids and flavonols such as metabolites in clade II in Section 3.1.3 such as vitexin, lonicerin, nicotiflorin, and rutin.

### 3.2. Transcriptome Differential Analysis

#### 3.2.1. Library Construction and Functional Analysis

This step illustrates the effectiveness of the filtration process. Subsequently, RNA-seq was performed on 12 samples, generating a total of 79.58 Gb of clean data, with an average of 6 Gb per sample. The Q30 percentage exceeded 92%, indicating high-quality sequencing data suitable for downstream gene expression analysis. To evaluate gene expression correlations, Pearson correlation coefficients were calculated for each sample pair and visualized in a heatmap. The Pearson correlation coefficient measures the similarity of gene expression levels between samples, with higher values indicating greater similarity. The analysis revealed high reproducibility among samples, as shown in Figure S5. Prior to analysis, raw sequencing reads were filtered to remove adapter contamination, low-quality reads, and reads with high proportions of unknown bases (N). Quality metrics of the filtered reads are provided in Table S2.

**Figure 5:**
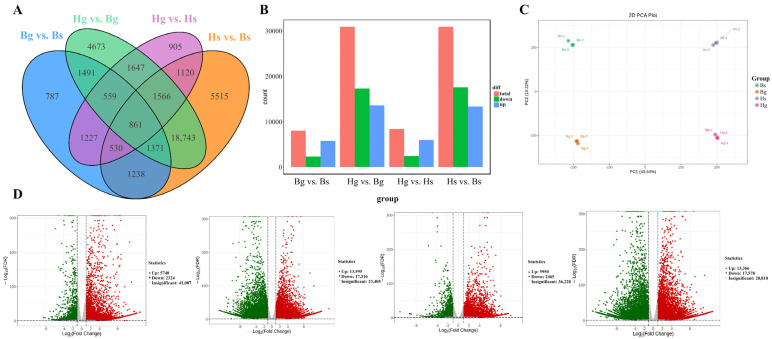
DEGs in the four comparisons. (**A**) Wayne diagram of differentially expressed genes; (**B**) rising/falling DEGs in three comparisons; (**C**) principal component analysis of gene expression; (**D**) volcano plots of DEGs from comparisons of Bg and Bs, Hg and Bg, Hg and Hs, and Hs and Bs (red/green dots indicating gene expression up/down, and gray dots indicating no difference; Hg: the disk flowers of HJ06; Hs: the ray flowers of HJ06; Bs: the disk flowers of BHYJ; Bg: the ray flowers of BHYJ. The dotted line represents the threshold for False Discovery Rate, denoting log_2_FC > 2, log_2_FC < −2, padj < 0.05, respectively).

A total of 861 common differentially expressed genes (DEGs) were identified across the Bs, Bg, Hs, and Hg samples (Figure 5A). In the bar graph, significantly differentially expressed genes are highlighted in red (upregulated) and blue (downregulated), as shown in Figure 5B. Principal component analysis (PCA) revealed distinct clustering patterns, with Hs, Hg, Bs, and Bg forming separate clusters, indicating significant differences among them (Figure 5C). Furthermore, the volcano plot visualizes differentially expressed genes (DEGs), with red indicating upregulation and green indicating downregulation, as shown in Figure 5D.

#### 3.2.2. Functional Analysis of DEGs

**Figure 6:**
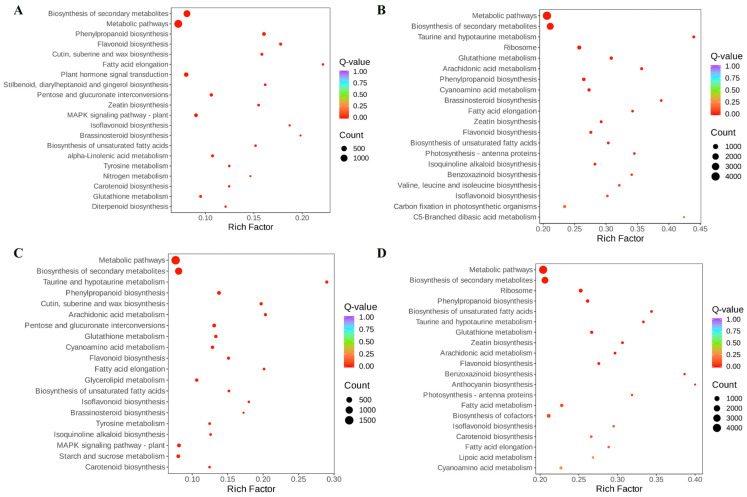
Diagram of KEGG enrichment of differentially expressed genes. (**A**) Comparison between Bg and Bs; (**B**) comparison between Hg and Bg; (**C**) comparison between Hg and Bg; (**D**) comparison between Hs and Bs (Hg: the disk flowers of HJ06; Hs: the ray flowers of HJ06; Bs: the disk flowers of BHYJ; Bg: the ray flowers of BHYJ).

To explore the functional roles of DEGs across genotypes, KEGG pathway enrichment analysis was performed on differentially expressed genes (DEGs) (Figure 6). In Bg, DEGs were significantly enriched in pathways including biosynthesis of secondary metabolites, plant hormone signal transduction, metabolic pathways, pentose and glucuronate interconversions, and MAPK signaling pathway-plant, compared to Bs. Similarly, DEGs in Hg were significantly enriched in pathways such as biosynthesis of secondary metabolites, ribosome, metabolic pathways, phenylpropanoid biosynthesis, and cyanoamino acid metabolism, compared to Bg. Furthermore, DEGs in Hg were significantly enriched in metabolic pathways, biosynthesis of secondary metabolites, MAPK signaling pathway-plant, phenylpropanoid biosynthesis, and starch and sucrose metabolism, compared to Hs. Finally, DEGs in Hs were significantly enriched in metabolic pathways, biosynthesis of secondary metabolites, biosynthesis of cofactors, ribosome, and phenylpropanoid biosynthesis, compared to Bs. DEGs enriched in the four sample groups were primarily associated with secondary metabolite biosynthesis, indicating significant differences in secondary metabolite production between BHYJ and HJ06. Interestingly, DEGs in the comparisons of Bg vs. Hg and Bs vs. Hs were significantly enriched in the phenylpropanoid biosynthesis pathway, suggesting differences in the upstream synthesis of flavonoid compounds between these sample pairs.

#### 3.2.3. Differential Gene and Key Enzyme Gene Selection

Following KEGG analysis, a total of 43 DEGs associated with the biosynthesis pathway of flavonoid compounds were identified. Among these, nine pathways were related to phenylpropanoid biosynthesis, including nine genes encoding *4CL*. Within the flavonoid biosynthesis pathway, 32 genes were identified, including 4 encoding *C4H*, 4 encoding *CHS*, 8 encoding *CHI*, 1 encoding *CHR*, 3 encoding *F3H*, 3 encoding DFR, 7 encoding *F3′H*, and 1 encoding *F3′5′H*. In the isoflavonoid biosynthesis pathway, one gene encoding *FNSII* was identified. The heatmap of key DEGs in the flavonoid biosynthesis pathway revealed that most genes were upregulated in ray flowers compared to disk flowers, and in HJ06 compared to BHYJ (Table S3).

### 3.3. Gene Co-Expression Network Analysis

**Figure 7:**
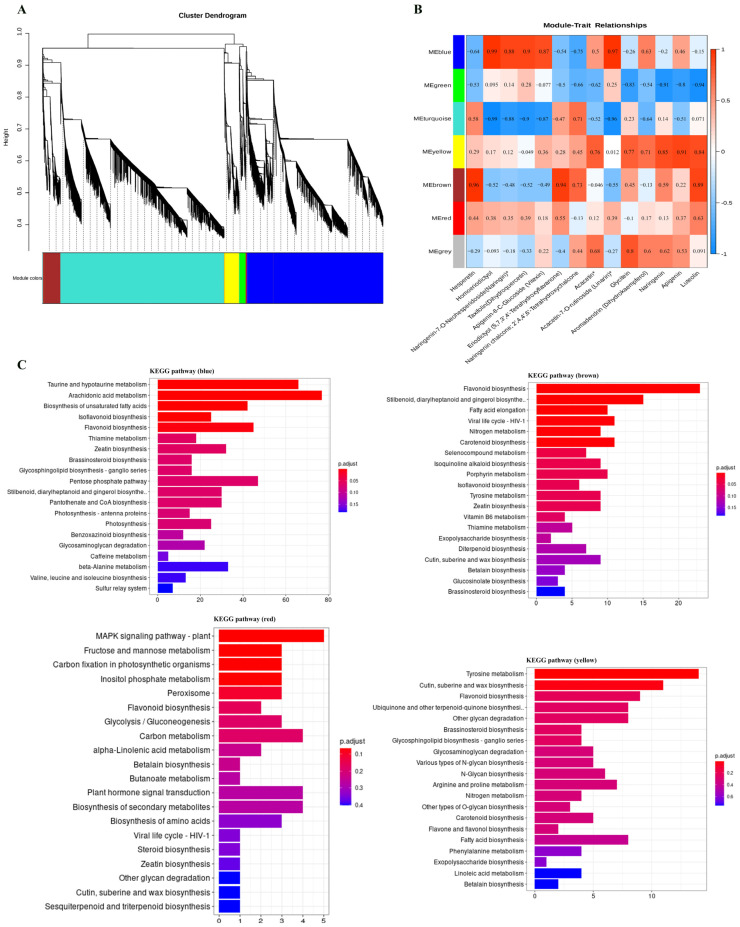
Co-expression network analysis. (**A**) Clustering dendrogram of DEGs; (**B**) module–trait associations. Each row corresponds to a module characteristic gene (eigengene), and each column corresponds to a trait. (**C**) KEGG enrichment analysis of genes within four modules (the four modules are blue, brown, red and yellow in that order). * indicates the compounds’ identification level was Level.

To identify flavonoid biosynthesis-related transcripts, we performed WGCNA on all DEGs, identifying seven distinct modules (Figure 7A). In addition to the gray module, which consisted of non-clustered genes, the blue, lime green, and brown modules contained the highest numbers of genes (11,815, 14,255, and 1526, respectively), while the red module contained the fewest genes (85) (Figure S5). Following WGCNA analysis, trait data were incorporated to generate a module–trait correlation heatmap, enabling further screening of candidate genes associated with changes in flavonoid composition. In the module–trait relationship analysis, the blue, yellow, brown, and red modules exhibited the highest correlation with the synthesis of most flavonoid metabolites (Figure 7B). As shown in the figure, genes significantly associated with linarin were located in the blue module, along with genes associated with the anabolic metabolism of homoeriodictyol. Subsequently, candidate genes were screened from the modules exhibiting the highest correlation.

Additionally, the KEGG library was employed to classify and annotate DEGs, further elucidating the biological functions of these four modules (Figure 7C). The results indicated that genes in the blue module were primarily associated with taurine and hypotaurine metabolism, arachidonic acid metabolism, and flavonoid biosynthesis. In the brown module, most genes were involved in flavonoid biosynthesis, while genes in the yellow module were mainly associated with tyrosine metabolism, cutin, suberine, and wax biosynthesis, as well as flavonoid biosynthesis. Notably, the brown module exhibited the highest percentage of genes involved in flavonoid biosynthesis, whereas the red module contained very few genes associated with this pathway. In summary, the brown module exhibited the highest percentage of genes involved in flavonoid biosynthesis, while the blue module contained the highest number of genes associated with this pathway, despite having the third-highest percentage. These findings further underscore the strong correlation between the blue and brown modules and flavonoid biosynthesis.

### 3.4. Integration Analysis of Metabolomics and Transcriptomics

**Figure 8:**
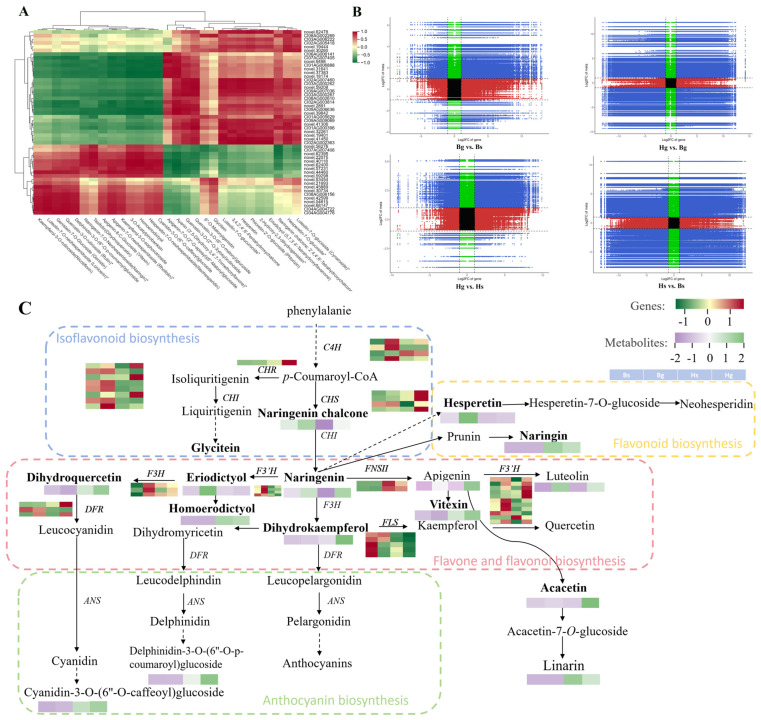
Joint metabolome and transcriptome analysis. (**A**) Correlation between flavone contents and differentially expressed flavonoid synthase gene expression; (**B**) nine quadrant plots between different samples; (**C**) regulatory network analysis of flavonoid accumulation (*n* = 3). * indicates the compounds’ identification level was Level.

To investigate the relationship between flavonoid compounds and key enzyme genes annotated in the transcriptomic data across different *C. indicum* varieties, a Pearson correlation analysis was performed by correlating the relative quantitative values of differential flavonoid compounds with the FPKM values of differentially expressed genes. The results were visualized using a heatmap. Most compounds, including 6″-*O*-malonylgenistin, glycitein, novel.56276, C107AG007406, novel.62395, novel.22075, novel.40118, novel.62400, novel.57231, and novel.44460, exhibited a positive correlation with genes, while showing a negative correlation with the remaining genes. Further details are provided in Figure 8A. To explore the specific correlations between genes and metabolites, a nine-quadrant chart was generated based on the fold changes of DAMs and DEGs (Figure 8B). In the comparison between disk and ray flowers, higher numbers of DAMs and DEGs were located in the third quadrant, indicating consistent expression patterns between genes and metabolites. In the comparison between BHYJ and HJ06, higher numbers of DAMs and DEGs were observed in the seventh and ninth quadrants. These components exhibited positive correlations in the seventh quadrant and negative correlations in the ninth quadrant.

In this study, based on the KEGG pathways related to flavonoid biosynthesis (ko00940, ko00941, ko00942, ko00943, and ko00944), we constructed regulatory network maps of SCMs and DEGs for BHYJ and HJ06, enriching them with 30 SCMs from the four major pathways and key enzyme genes identified previously. As shown in Figure 8C, compounds such as naringenin chalcone, hesperetin, and eriodictyol exhibited high expression in BHYJ, while naringenin showed high expression in disk flowers. Vitexin, acacetin, linarin, and dihydrokaempferol were highly expressed in HJ06, with the overall pattern indicating higher expression of compounds in the linarin metabolic pathway in HJ06 compared to BHYJ. Some DEGs exhibited trends consistent with downstream metabolites, such as the regulatory trends of *F3′H* and *FNSII*, which aligned with the expression changes of compounds like eriodictyol and vitexin. In contrast, the expression of *F3H*-related genes and the accumulation of their downstream products, such as dihydroquercetin and dihydrokaempferol, exhibited opposite trends. No obvious correlation was observed between other DEGs and their downstream SCMs, likely due to the formation of a complex regulatory network involving multi-substance and multi-gene interactions in flavonoid synthesis and expression in *C. indicum*, rather than a simple one-to-one correspondence between metabolites and genes.

### 3.5. Verification of Key Enzyme Genes

**Figure 9:**
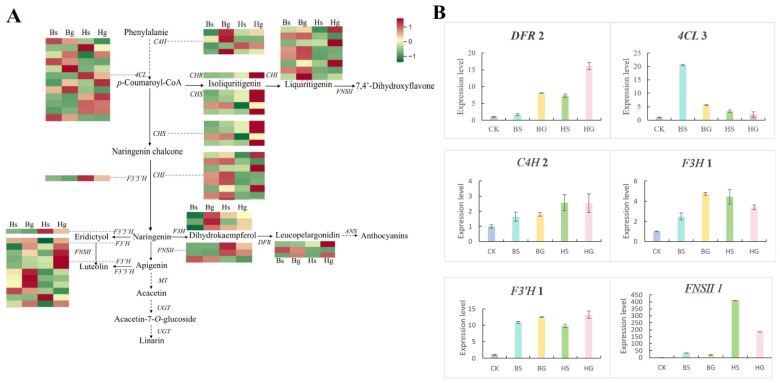
Expression patterns of flavonoid biosynthesis genes. (**A**) Heatmap of the expression levels of genes in flavonoid biosynthesis; (**B**) the relative expression levels of some genes in flavonoid biosynthesis.

To characterize the expression dynamics of flavonoid-related genes, a comprehensive analysis was conducted on genes involved in the flavonoid biosynthesis pathway in *C. indicum*. Transcriptome sequencing analysis revealed significant differential expression of genes in this pathway (Figure 9A). Specifically, downstream naringenin derivatives, including hesperetin and eriodictyol, were more abundant in BHYJ than in HJ06. Concurrently, key enzyme genes in this pathway, particularly *F3H* and *F3′H*, exhibited higher expression levels in BHYJ. In contrast, compounds associated with linarin accumulation, such as vitexin and acacetin, showed higher abundance in HJ06, with the corresponding enzyme gene FNSII demonstrating a similar expression pattern. To validate the differentially expressed genes (DEGs) identified by Illumina sequencing, six key genes in the flavonoid biosynthetic pathway were selected for RT-qPCR analysis. RT-qPCR results confirmed that the six selected genes exhibited distinct expression patterns across Bs, Bg, Hs, and Hg samples, consistent with the RNA-Seq results shown in Figure 9B.

## 4. Discussion

### 4.1. Elucidating Flavonoid Synthesis Pathways in BHYJ and HJ06

The capitulum of *C. indicum* is commonly yellow [29]. However, a rare white ray flower variant of *C. indicum* lacking linarin was identified in this study. This discovery raises the following question: at which stage of the flavonoid biosynthetic pathway is linarin production affected? Further investigation is warranted. Comprehensive transcriptomic and metabolomic analyses [30] enable the elucidation of biosynthetic pathways and metabolic regulation mechanisms in differently pigmented *C. indicum* varieties, offering valuable insights for optimizing linarin content and quality through integrated systems biology and metabolic engineering approaches [31]. In this study, we selected HJ06 and BHYJ as representative cultivars, systematically analyzing both disk and ray florets to investigate spatial variations in gene expression patterns and metabolite accumulation profiles in *C. indicum*.

Our findings demonstrate organ-specific yet consistent expression patterns of flavonoid components across both cultivars, aligning with previous reports [32]. Quantitative analysis revealed significantly elevated levels of specific flavonoid metabolites in HJ06, including naringenin-4′-*O*-glucoside, vitexin-2″-*O*-galactoside, luteolin-4′-*O*-glucoside, and quercetin-3-*O*-rutinoside-7-*O*-glucoside, compared to BHYJ. A total of 30 metabolites associated with flavonoid biosynthetic pathways were identified and quantified in both HJ06 and BHYJ cultivars. Notably, compounds such as naringin, dihydroquercetin, lonicerin, nicotiflorin, rhoifolin, and neohesperidin exhibited significantly higher expression levels in HJ06, whereas metabolites including eriodictyol, ayanin, and naringenin chalcone were predominantly accumulated in BHYJ. Therefore, it is not recommended to assess the suitability of BHYJ as a cultivar based solely on differential metabolites. According to the Chinese Pharmacopoeia, the specified indicator component is the content of linarin [33]; the level of linarin in HJ06 is significantly higher than in BHYJ, suggesting the need for further investigation into the medicinal value of BHYJ. The theory of “identifying symptoms and discussing quality” posits that the fixed characteristics expressed in the appearance of herbs, such as “shape, color, odors, taste, and texture”, have a certain relationship with their internal qualities [34]. With the development of science and technology combined with the spectrophotometer, the electronic tongue, mass spectrometry, and other technologies, trait data and chemical composition are associated with the promotion of “empirical identification of traits” to “objective identification of traits” [35]. Bitterness as a characteristic trait of *C. indicum* varies significantly in different germplasms, and in this paper, two materials were analyzed by electronic tongue analysis to explore the medicinal and edible qualities of BHYJ and HJ06 using bitterness as an entry point. The results showed that the bitterness of both disk and ray flowers of BHYJ was lower than that of HJ06, and correlation analysis with the differential metabolites hypothesized that the reduction in the accumulation of flavonoids and flavonols, such as linarin, rhoiflolin, vitexin, rutin, nicotiflorin, etc., in BHYJ caused a reduction in the bitterness of *C. indicum*. Metabolites functioning as precursors or intermediates in the flavonoid synthesis pathways can significantly influence the production of downstream flavonoid compounds. Naringenin chalcone, a key intermediate in the flavonoid pathway, is partitioned into various pathways to form different flavonoids. The content of naringenin chalcone is higher in BHYJ than in HJ06. However, due to its greater partitioning toward the accumulation of hesperetin, eriodictyol, and dihydroquercetin in BHYJ, the remaining naringenin available for linarin accumulation is reduced. Consequently, the levels of vitexin, apigenin, and acacetin are higher in HJ06 than in BHYJ, consistent with this trend. It was hypothesized that the differential allocation of naringenin into various synthesis pathways might account for the observed differences in monensin content between the two *C. indicum* species. Meanwhile, the expression levels of key enzyme genes regulating the metabolism of naringenin to eriodictyol and dihydroquercetin were higher in BHYJ than in HJ06. In contrast, the expression levels of key enzyme genes regulating the metabolism of naringenin to apigenin, vitexin, and acacetin were lower in BHYJ. Additionally, the synthesis of key intermediates in the monensin synthesis pathway was lower in BHYJ than in HJ06, while the consumption of these intermediates was higher. These factors may collectively explain the higher linarin content in HJ06 compared to BHYJ.

### 4.2. Unraveling the Key Enzyme Genes in Flavonoid Biosynthesis Pathways of C. indicum Through Integrated Transcriptomics and Metabolomics Analysis

The integration of metabolomics and transcriptomics analyses has been widely utilized to elucidate the response mechanisms associated with secondary metabolite accumulation, representing a robust technological approach for identifying potential key regulatory genes [36]. For example, Zhang et al. employed an integrated metabolomics–transcriptomics approach to elucidate the molecular regulatory networks and identify candidate key genes associated with differential flavonoid accumulation across various organs in two *Lonicera macranthoides* varieties [37].

To date, the exploration and functional characterization of key enzymatic genes involved in flavonoid biosynthetic pathways in *C. indicum* remain insufficient, with current research primarily focused on single varieties, resulting in limited genetic diversity among study subjects. In this study, we leveraged the synergistic advantages of integrated metabolomics and transcriptomics analyses to identify key enzymatic genes involved in flavonoid biosynthesis, focusing on the differential accumulation patterns of flavonoid components between BHYJ and HJ06 varieties across various organs [38]. Through comprehensive integration of transcriptomic and metabolomic analyses coupled with spatiotemporal expression profiling, we identified several critical enzymatic genes, including chalcone isomerase (*CHI*) [39], *Flavonoid 3′-hydroxylase* (*F3′H*) [40], *Flavanone 3-hydroxylase* (*F3H*) [41], *Flavone synthase II* (*FNSII*) [42], and *Dihydroflavonol-4-reductase* (*DFR*) [37], which potentially regulate flavonoid accumulation in BHYJ and HJ06, demonstrating significant interspecific and organ-specific expression patterns. *CHI* accelerates the isomerization reaction, facilitating the conversion of chalcone to naringenin. Sun et al. demonstrated that CHI serves as a key enzyme in the biosynthesis of anthocyanins in *Ophiorrhiza japonica* [43]. F3H functions as a pivotal regulatory hub in the flavonoid metabolic pathway, controlling the metabolic flux throughout the biosynthetic route [41]. As a member of the cytochrome P450 monooxygenase family, F3′H catalyzes the hydroxylation at the *C*-3′ position of the B-ring in flavonoid molecules. FNS II catalyzes the 2,3-desaturation of flavanones, resulting in the formation of flavones through the introduction of a double bond between C-2 and C-3 positions [44].

Our investigation revealed a significant variation in linarin content between BHYJ and HJ06 cultivars. Metabolic flux analysis suggests that the divergence occurs at the naringenin node, where elevated expression levels of *F3H* and *F3′H* in BHYJ, compared to HJ06, direct the conversion of naringenin to eriodictyol and dihydrokaempferol. This metabolic shift results in reduced naringenin availability, consequently limiting linarin biosynthesis. The *FNS II* gene exhibits high expression levels in HJ06, thereby significantly regulating the conversion of naringin to apigenin, which leads to increased accumulation toward linarin synthesis. The differential accumulation of flavonoid components between BHYJ and HJ06 appears to be primarily regulated by the expression patterns of upstream genes in the flavonoid biosynthesis pathway. Modulation of these regulatory genes through either upregulation of upstream genes to enhance overall flavonoid accumulation or inhibition of branch-specific genes to promote linarin biosynthesis represents a potential strategy for metabolic engineering. Future research directions may include the following: (1) targeted pathway modification through CRISPR/Cas9-mediated gene editing to eliminate competing metabolic branches [45], (2) introduction of key biosynthetic or regulatory genes, and (3) development of elite *C. indicum* germplasm as genetic resources for cultivar improvement.

### 4.3. Flower Color Variations from a Medicinal and Eatable Perspective

In medicinal plant research, flower color variation is closely associated with phytochemical composition, which directly influences both medicinal properties and culinary value. Significant phytochemical variations exist among differently colored flowers, particularly in secondary metabolites including flavonoids, anthocyanins, fatty acids, and amino acids [46]. Beyond influencing flower pigmentation, these chemical variations affect fragrance profiles and medicinal efficacy. For instance, Ma et al. demonstrated that white-flowered licorice variants exhibit elevated glycyrrhizic acid content, establishing a correlation between flower color and bioactive compound accumulation [47]. This observation aligns with traditional medicinal practices that utilize color as an indicator of herbal quality. Comparative analysis of cyclic enol ether terpenoid glycosides in blue-violet versus white flowers of the Tibetan medicinal plant Jie-Ji revealed distinct chemical profiles, further supporting the correlation between flower color and phytochemical composition [48]. Color-component correlation analysis of *Lonicera japonica* Thunb. buds identified significant associations between flower color and specific bioactive compounds, including rutin and isochlorogenic acid B, potentially serving as phytochemical markers for quality assessment [49].

Therefore, the present study focused on exploring the differences in flavonoid composition and their molecular mechanisms that accompany changes in flower color. Significant phytochemical differences exist between yellow and white capitula of *C. indicum*, particularly in flavonoid composition, including linarin, which potentially influences both organoleptic properties and pharmacological activity [15,50]. Wild-type *C. indicum* typically produces yellow capitula [51], with this phenotype being predominant in natural populations. Cultivation conditions, including environmental factors, can significantly influence capitulum pigmentation. In the same genus of plants, medicinal chrysanthemums exhibit color variations ranging from yellow to white, which are commonly observed. Contrary to initial assumptions, recent studies have demonstrated that flavonoid profiles do not directly contribute to color variation in *C. indicum* [1]. Emerging evidence indicates that carotenoid accumulation patterns, regulated by differential expression of carotenoid cleavage dioxygenase (CmCCD4a), primarily determine yellow–white color variation in *Chrysanthemum morifolium* petals [52]. Although pigment biosynthesis pathways differ from other secondary metabolite pathways, these metabolic networks are interconnected, exhibiting coordinated regulation during floral development and in response to environmental stresses [53]. Consequently, the interdependent relationship between color variation and flavonoid metabolism in *C. indicum* requires a systems-level approach, reflecting the inherent complexity of studying bioactive compound dynamics in medicinal and food plants. Elucidating the regulatory mechanisms underlying flavonoid variation in *C. indicum* serves as a crucial link connecting phenotypic traits, phytochemical composition, and biological efficacy, while providing essential insights for the sustainable development and utilization of medicinal and food resources.

## 5. Conclusions

In this study, a rare white *C. indicum* without linarin was found for the first time, which provided an opportunity to explore the regulation mechanism of flavonoid metabolism. The comparative analysis of transcriptomic and metabolomic data of HJ06 and BHYJ revealed the differences in the regulation of flavonoid synthesis in different-colored *C. indicum*. Metabolic profiling showed that the organ distribution patterns of flavonoid components were similar in both species, but the contents of key intermediates showed significant differences: naringenin was higher in HJ06, while downstream products such as saccharin were more enriched in BHYJ; vitexin and acacetin were more predominant in HJ06. The difference in the diversion of naringenin as a metabolic hub resulted in the different accumulation patterns of flavonoids in the two varieties. Transcriptome analysis showed that the gene expression activity was higher in HJ06 than in BHYJ, and was higher in ray flowers than in disk flowers. The joint multi-omics study elucidated the regulation mechanism of the flavonoid metabolism network and provided a theoretical basis for the targeted regulation of linarin synthesis. In the future, we can optimize the allocation of metabolic fluxes through gene editing technology and cultivate high-quality *C. indicum* varieties.

## Figures and Tables

**Table 1:** Identification of flavonoid compounds in *Chrysanthemum indicum*.

No.	Q1 (Da)	Q3 (Da)	Molecular Weight (Da)	Formula	Ionization Model	Compounds	CAS	Level
1	287	125	288	C_15_H_12_O_6_	[M-H]^−^	2-Hydroxy-2,3-dihydrogenistein *	-	2
2	519	271	518	C_24_H_22_O_13_	[M+H]^+^	6″-O-Malonylgenistin	51011-05-3	1
3	431	269	432	C_21_H_20_O_10_	[M-H]^−^	Genistein-7-O-Glucoside (Genistin)	529-59-9	2
4	285	242	284	C_16_H_12_O_5_	[M+H]^+^	Glycitein	40957-83-3	2
5	289	163	288	C_15_H_12_O_6_	[M+H]^+^	3,4,2′,4′,6′-Pentahydroxychalcone	73692-51-0	2
6	313	253	314	C_17_H_14_O_6_	[M-H]^−^	3-O-Acetylpinobanksin	52117-69-8	3
7	431	311	432	C_21_H_20_O_10_	[M-H]^−^	Apigenin-8-C-Glucoside (Vitexin)	3681-93-4	1
8	287	135	288	C_15_H_12_O_6_	[M-H]^−^	Eriodictyol (5,7,3′,4′-Tetrahydroxyflavanone)	552-58-9	1
9	301	164	302	C_16_H_14_O_6_	[M-H]^−^	Hesperetin	520-33-2	1
10	465	303	464	C_22_H_24_O_11_	[M+H]^+^	Hesperetin-7-O-glucoside *	31712-49-9	3
11	611	303	610	C_28_H_34_O_15_	[M+H]^+^	Hesperetin-7-O-neohesperidoside (Neohesperidin)	13241-33-3	3
12	303	153	302	C_16_H_14_O_6_	[M+H]^+^	Homoeriodictyol	446-71-9	2
13	273	153	272	C_15_H_12_O_5_	[M+H]^+^	Naringenin chalcone; 2′,4,4′,6′-Tetrahydroxychalcone	73692-50-9	3
14	579	271	580	C_27_H_32_O_14_	[M-H]^−^	Naringenin-7-O-Neohesperidoside (Naringin) *	10236-47-2	3
15	435	167	436	C_21_H_24_O_10_	[M-H]^−^	Phloretin-2′-O-glucoside (Phlorizin)	60-81-1	3
16	303	125	304	C_15_H_12_O_7_	[M-H]^−^	Taxifolin (Dihydroquercetin)	480-18-2	3
17	651	271	650	C_29_H_30_O_17_	[M+H]^+^	Apigenin-7-O-(2″-O-apiosyl)(6′’-Malonyl)glucoside	-	3
18	579	271	578	C_27_H_30_O_14_	[M+H]^+^	Apigenin-7-O-neohesperidoside (Rhoifolin) *	17306-46-6	1
19	431	311	432	C_21_H_20_O_10_	[M-H]^−^	Apigenin-8-C-Glucoside (Vitexin)	3681-93-4	1
20	345	330	344	C_18_H_16_O_7_	[M+H]^+^	Ayanin (3′,5-Dihydroxy-3,4′,7-Trimethoxyflavone) *	572-32-7	1
21	593	285	594	C_27_H_30_O_15_	[M-H]^−^	Kaempferol-3-O-rutinoside (Nicotiflorin)	17650-84-9	2
22	773	449	772	C_33_H_40_O_21_	[M+H]^+^	Kaempferol-3-O-sophorotrioside	80714-53-0	2
23	449	287	448	C_21_H_20_O_11_	[M+H]^+^	Luteolin-7-O-glucoside (Cynaroside) *	5373-11-5	1
24	463	287	462	C_21_H_18_O_12_	[M+H]^+^	Luteolin-7-O-glucuronide *	29741-10-4	1
25	595	287	594	C_27_H_30_O_15_	[M+H]^+^	Luteolin-7-O-neohesperidoside (Lonicerin) *	25694-72-8	1
26	743	449	742	C_32_H_38_O_20_	[M+H]^+^	Quercetin-3-O-(2″-O-Xylosyl)rutinoside	129235-39-8	3
27	549	300	550	C_24_H_22_O_15_	[M-H]^−^	Quercetin-3-O-(6″-O-malonyl)glucoside	96862-01-0	3
28	611	303	610	C_27_H_30_O_16_	[M+H]^+^	Quercetin-3-O-rutinoside (Rutin) *	153-18-4	1
29	611	287	611	C_30_H_27_O_14_^+^	[M]^+^	Cyanidin-3-O-(6″-O-caffeoyl)glucoside	-	3
30	611	303	611	C_30_H_27_O_14_^+^	[M]^+^	Delphinidin-3-O-(6″-O-p-coumaroyl)glucoside	-	3

No: Number. Q1: Parent ion. Q3: Product ion. *: Correction of standard. Level: Qualitative level, higher is better, 3 > 2 > 1.

## Data Availability

All data generated or analyzed during this study are included in this manuscript and its Supplementary Information Files. All data in the manuscript have been uploaded to Figureshare (https://figshare.com). The DOI number of the data is (10.6084/m9.figshare.28028273).

## Supplementary Materials

The following supporting information can be downloaded at: https://www.mdpi.com/article/10.3390/foods14111896/s1, Table S1. Details of genes and primers involved in RT-qPCR. Table S2. Quality statistics of filtered reads. Table S3. Differentially expressed genes associated with flavonoid. Figure S1. Determination of linarin content in BSYJ. Figure S2. MRM detection of multimodal maps. Figure S3. Differential flavonoid metabolite in BSYJ and HJ06. Figure S4. Histogram of BHYJ and HJ06 electronic tongue data. Figure S5. Pearson correlation analysis of all samples. Figure S6. Module group trait correlation.
