# Supplementary material for: Decoding Flavonoid Metabolism for Nutritional Enhancement: A Transcriptome–Metabolome Integration Study of Biosynthesis in Edible Chrysanthemum indicum L
Source: Foods. 2025 May 26;14(11):1896. doi: 10.3390/foods14111896 (PMC12155382; doi:10.3390/foods14111896)
Supplement: Supplementary file 1 [file foods-14-01896-s001.zip › foods-3605012-supplementary.pdf]

**Supplementary materials**

**Table S1.** Details of genes and primers involved in RT-qPCR.

**Table S2.** Quality statistics of filtered reads.

**Table S3.** Differentially expressed genes associated with flavonoid.

**Figure S1.** Determination of linarin content in BSYJ.

**Figure S2.** MRM detection of multimodal maps.

**Figure S3.** Differential flavonoid metabolite in BSYJ and HJ06.

**Figure S4.** Histogram of BHYJ and HJ06 electronic tongue data.

**Figure S5.** Pearson correlation analysis of all samples.

**Figure S6.** Module group trait correlation.

**Table S1. Details of genes and primers involved in RT-qPCR**

| Gene number | F (5'→3') | R (5'→3') |
|---|---|---|
| *DFR-2 (novel.68174)* | AAGAAGACTCACCAGCCATTGTAT | TCCGCCTTCCATAATGTCAAGT |
| *4CL-3 (novel.18174)* | GAGTGTGCTAAGAAGTTCCCTCA | TCAAGTCCTGGAATAAGCCTTCC |
| *C4H-2 (novel.40118)* | GAGGCAGCAGCAGTTGTG | AGTCTTCTTCTTAGCACGATACCT |
| *F3H-1 (novel.27976)* | GGTGTTGTCCGAGGCAATG | AACTTGGTCCTGAAGCAATAACG |
| *F3'H-1 (novel.21693)* | CGGCTTGTAACAGAATTGGACTTG | GGTAAGGAGAGTGGCGTTGAA |
| *FNSII-1 (novel.62400)* | TCAACTCACTATGGTCCGCTAATC | AAGGAAGTCACGAGCAAGGT |

**Table S2. Quality statistics of filtered Reads**

| Sample | Total Raw Reads （M） | Total Clean Reads （M） | Total Clean Bases （Gb） | Clean Reads Q20 （%） | Clean Reads Q30 （%） | GC Content （%） |
|---|---|---|---|---|---|---|
| Bs-1 | 45973020 | 44569460 | 6.69 | 98.36 | 94.91 | 42.35 |
| Bs-2 | 46951188 | 45030066 | 6.75 | 98.65 | 95.7 | 42.56 |
| Bs-3 | 44601880 | 42901630 | 6.44 | 98.62 | 95.57 | 42.55 |
| Bg-1 | 48089546 | 46608760 | 6.99 | 98.61 | 95.53 | 42.51 |
| Bg-2 | 43095894 | 42099838 | 6.31 | 98.51 | 95.31 | 42.57 |
| Bg-3 | 52792028 | 51183122 | 7.68 | 98.62 | 95.6 | 42.54 |
| Hs-1 | 42717612 | 41290056 | 6.19 | 98.66 | 95.71 | 42.71 |
| Hs-2 | 49201448 | 47616334 | 7.14 | 98.53 | 95.35 | 42.7 |
| Hs-3 | 44831260 | 43334396 | 6.5 | 98.58 | 95.5 | 42.72 |
| Hg-1 | 44460202 | 42658142 | 6.4 | 98.58 | 95.47 | 42.67 |
| Hg-2 | 45393478 | 42843108 | 6.43 | 98.72 | 95.83 | 42.68 |
| Hg-3 | 49326404 | 47325750 | 7.1 | 98.5 | 95.28 | 42.66 |

**Table S3. Differentially expressed genes associated with flavonoid**

| Gene ID | Name | KEGG Orthology | KEGG | Bg vs Bs | Hg vs Bg | Hg vs Hs | Hs vs Bs |
|---|---|---|---|---|---|---|---|
| CI03AG006222 | C4H | K00487 | ko00130 | up | | | |
| CI09AG007036 | C4H | K00487 | ko00130 | | down | | down |
| novel.40118 | C4H | K00487 | ko00130 | | up | | up |
| novel.62478 | C4H | K00487 | ko00130 | up | down | | |
| novel.18174 | 4CL | K01904 | ko00130 | | down | | down |
| novel.19401 | 4CL | K01904 | ko00130 | up | down | | |
| novel.22075 | 4CL | K01904 | ko00130 | | up | | up |
| novel.28225 | 4CL | K01904 | ko00130 | up | | | |
| novel.2891 | 4CL | K01904 | ko00130 | | up | up | |
| novel.44460 | 4CL | K01904 | ko00130 | | up | | up |
| novel.57231 | 4CL | K01904 | ko00130 | | up | | up |
| novel.59298 | 4CL | K01904 | ko00130 | | up | | up |
| novel.9498 | 4CL | K01904 | ko00130 | | down | | down |
| novel.68147 | CHS | K00660 | ko00941 | up | up | up | up |
| CI04AG004722 | CHS | K00660 | ko00941 | up | up | up | up |
| CI01AG005629 | CHS | K00660 | ko00941 | | | up | dwon |
| novel.45889 | CHS | K00660 | ko00941 | | up | up | |
| novel.53454 | CHI | K01859 | ko00941 | | | up | |
| CI02AG003814 | CHI | K01859 | ko00941 | | down | | down |
| CI08AG006156 | CHI | K01859 | ko00941 | up | up | up | up |
| CI09AG006636 | CHI | K01859 | ko00941 | | down | | down |

| | | | | | | | |
|---|---|---|---|---|---|---|---|
| CI09AG008109 | CHI | K01859 | ko00941 | | down | | down |
| novel.30734 | CHI | K01859 | ko00941 | up | up | up | |
| novel.39842 | CHI | K01859 | ko00941 | | down | | down |
| novel.41450 | CHI | K01859 | ko00941 | up | down | | |
| novel.56276 | CHR | K08243 | ko00941 | | up | down | up |
| novel.62395 | FNSII | K23179 | ko00941 | | up | | up |
| novel.62400 | FNSII | K23179 | ko00941 | | up | | up |
| CI01AG006888 | FNSII | K23179 | ko00941 | | down | | down |
| CI08AG001748 | F3H | K00475 | ko00941 | up | | | up |
| novel.19444 | F3H | K00475 | ko00941 | up | | | |
| novel.27976 | F3H | K00475 | ko00941 | up | | | up |
| CI04AG004776 | DFR | K13082 | ko00941 | | up | up | up |
| novel.37383 | DFR | K13082 | ko00941 | | down | down | down |
| novel.68174 | DFR | K13082 | ko00941 | up | | up | |
| novel.25052 | F3'5'H | K13083 | ko00941 | | | up | up |
| CI07AG006872 | F3'H | K05280 | ko00941 | | | up | down |
| novel.21679 | F3'H | K05280 | ko00941 | up | | | |
| novel.21693 | F3'H | K05280 | ko00941 | | | up | |
| novel.42599 | F3'H | K20770 | -- | | up | up | |
| novel.54615 | F3'H | K20770 | -- | up | up | up | up |
| CI01AG000396 | F3'H | K20770 | -- | | down | up | down |
| CI02AG007460 | F3'H | K20770 | -- | up | down | up | down |

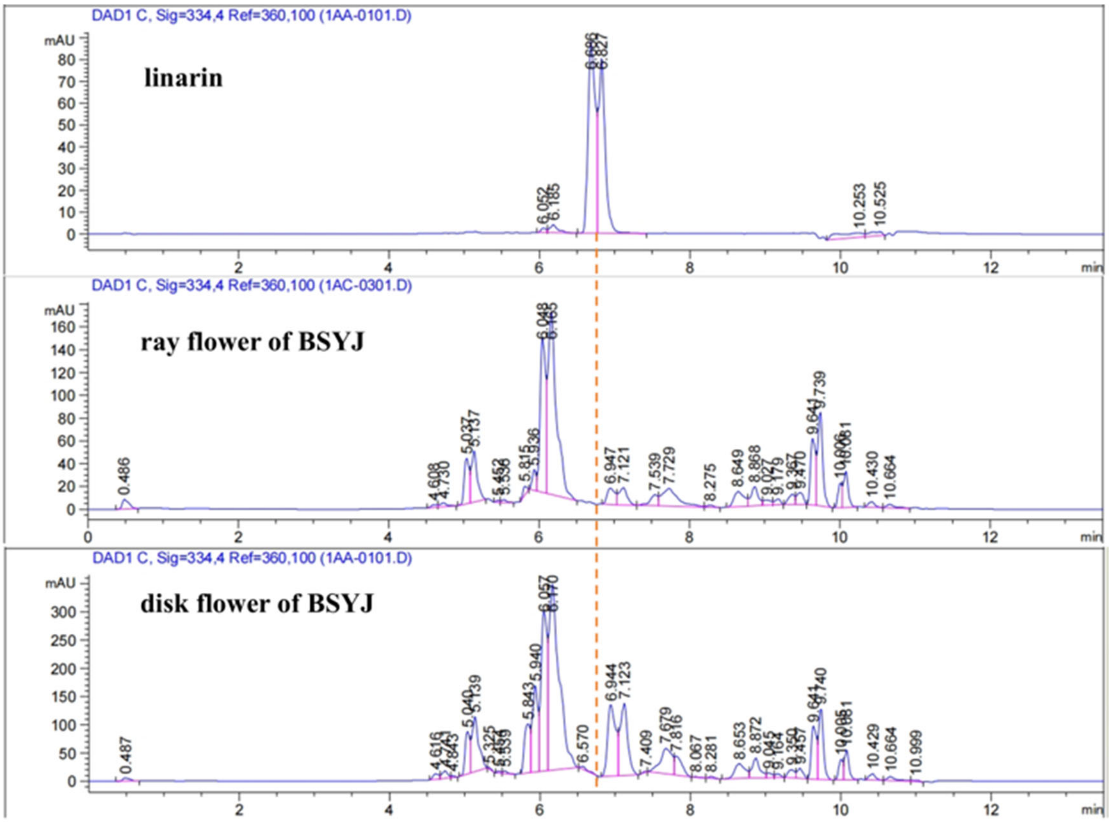


**Figure S1. Determination of linarin content in BSYJ.**

A

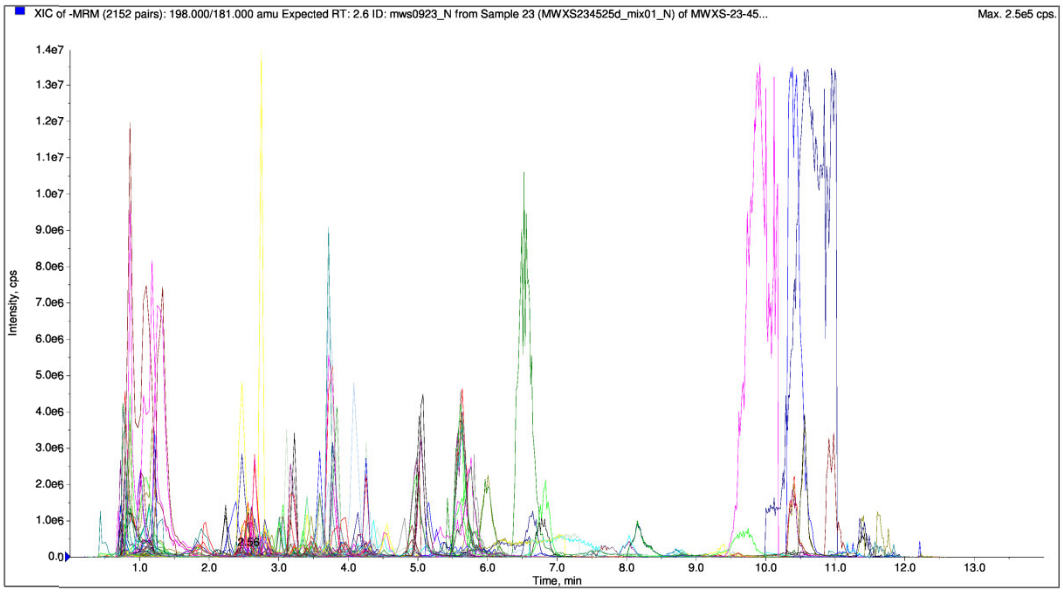


B

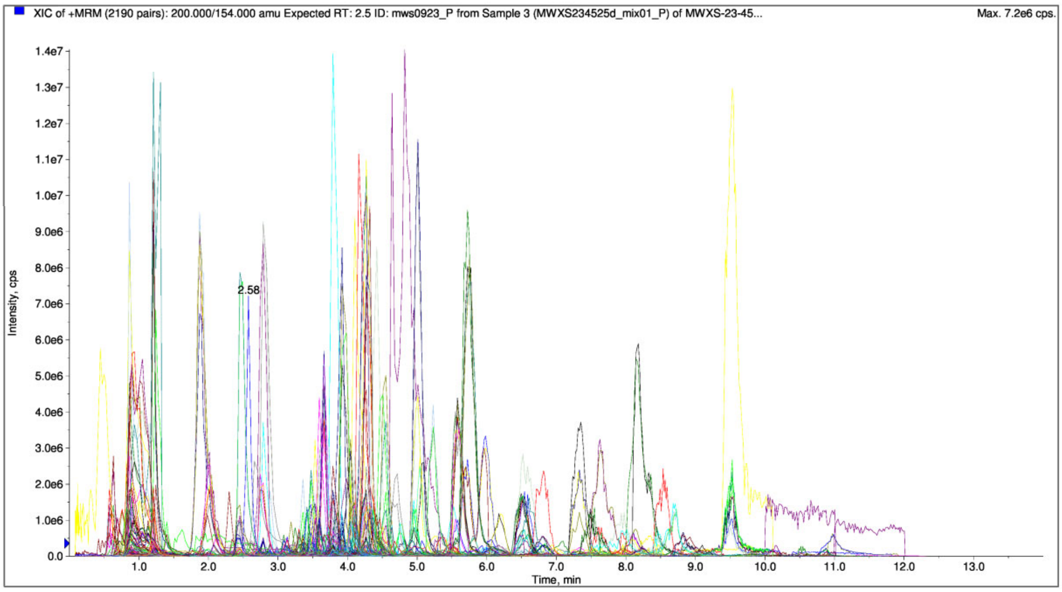


**Figure S2. MRM detection of multimodal maps**

(A) MWXS-23-4525-d_MRM_detection_of_multimodal_maps-Negative

(B) MWXS-23-4525-d_MRM_detection_of_multimodal_maps-Positive

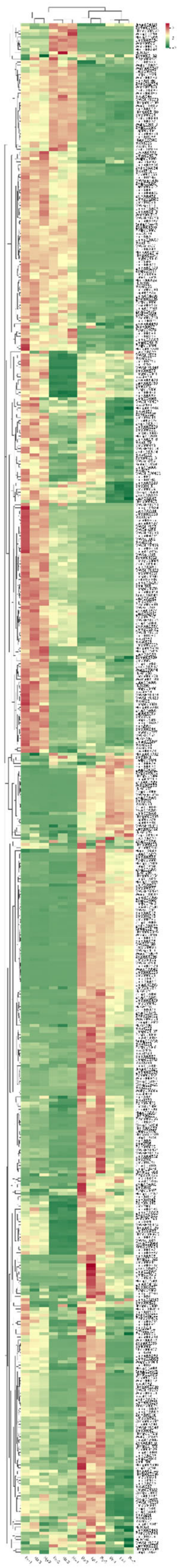

**Figure S3.** Differential flavonoid metabolite in BSYJ and HJ06.

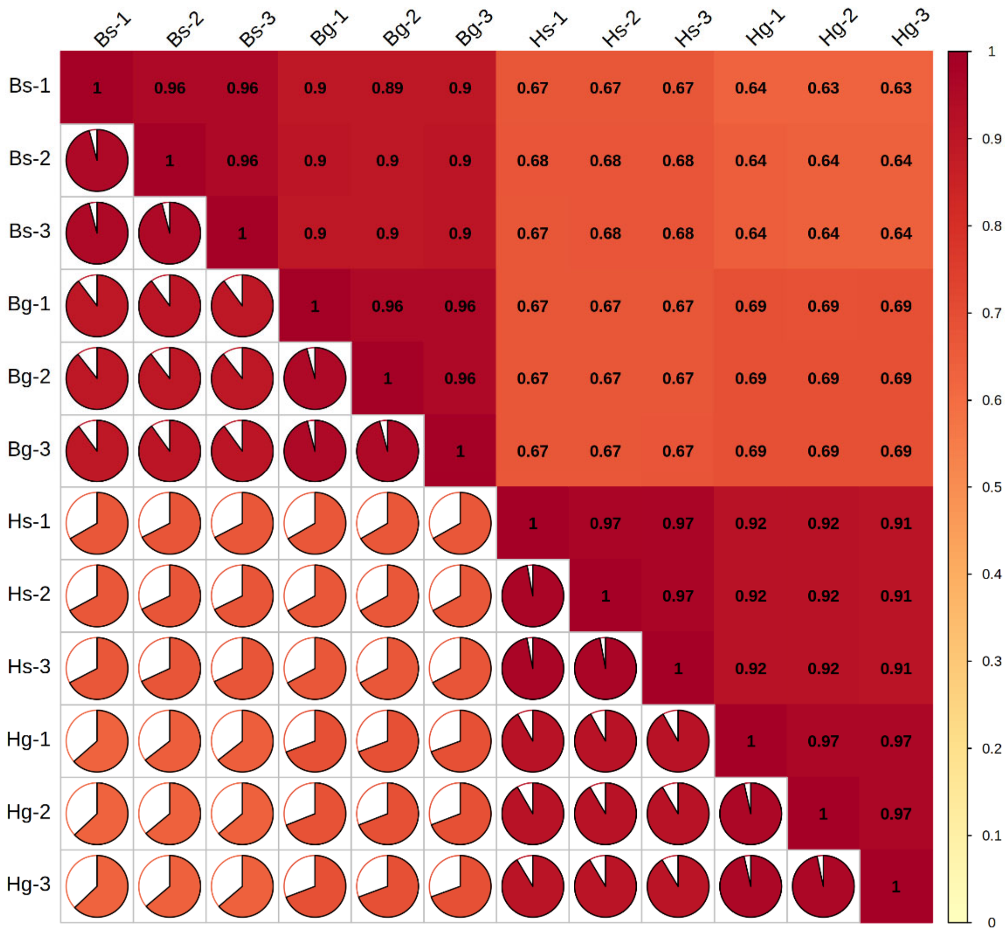


**Figure S4. Pearson correlation analysis of all samples**

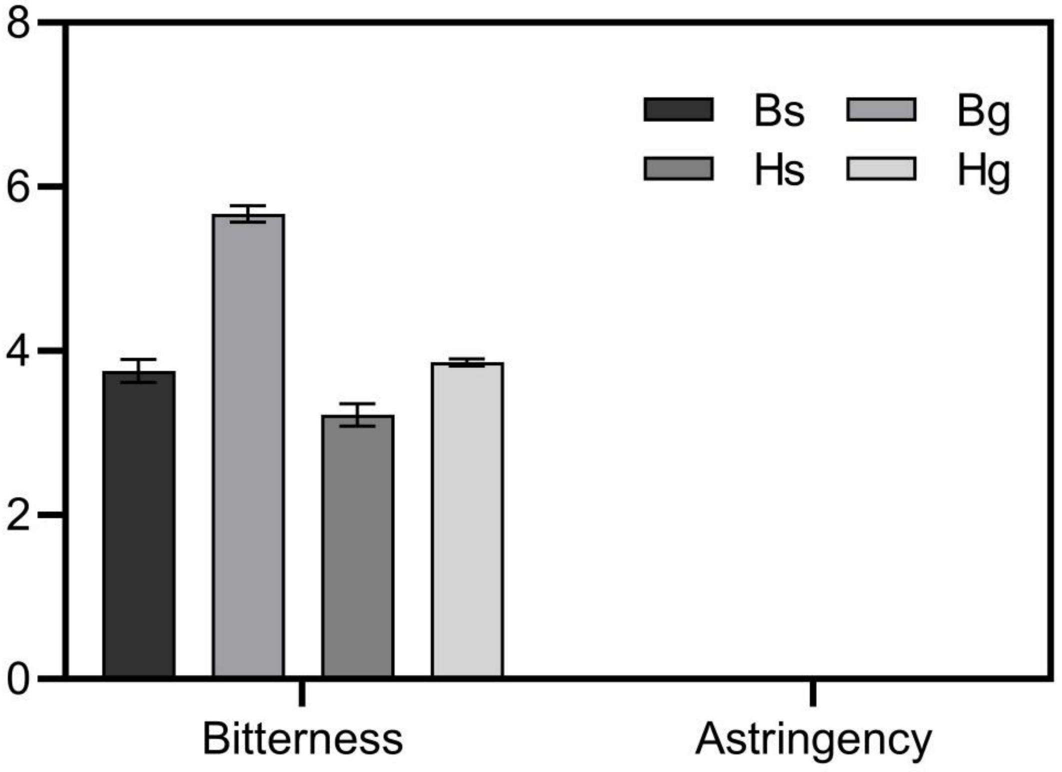


**Figure S5.** Pearson correlation analysis of all samples

Hg: the disk flowers of HJ06，Hs: the ray flowers of HJ06, Bs: the disk flowers of BHYJ , Bg: the ray flowers of BHYJ

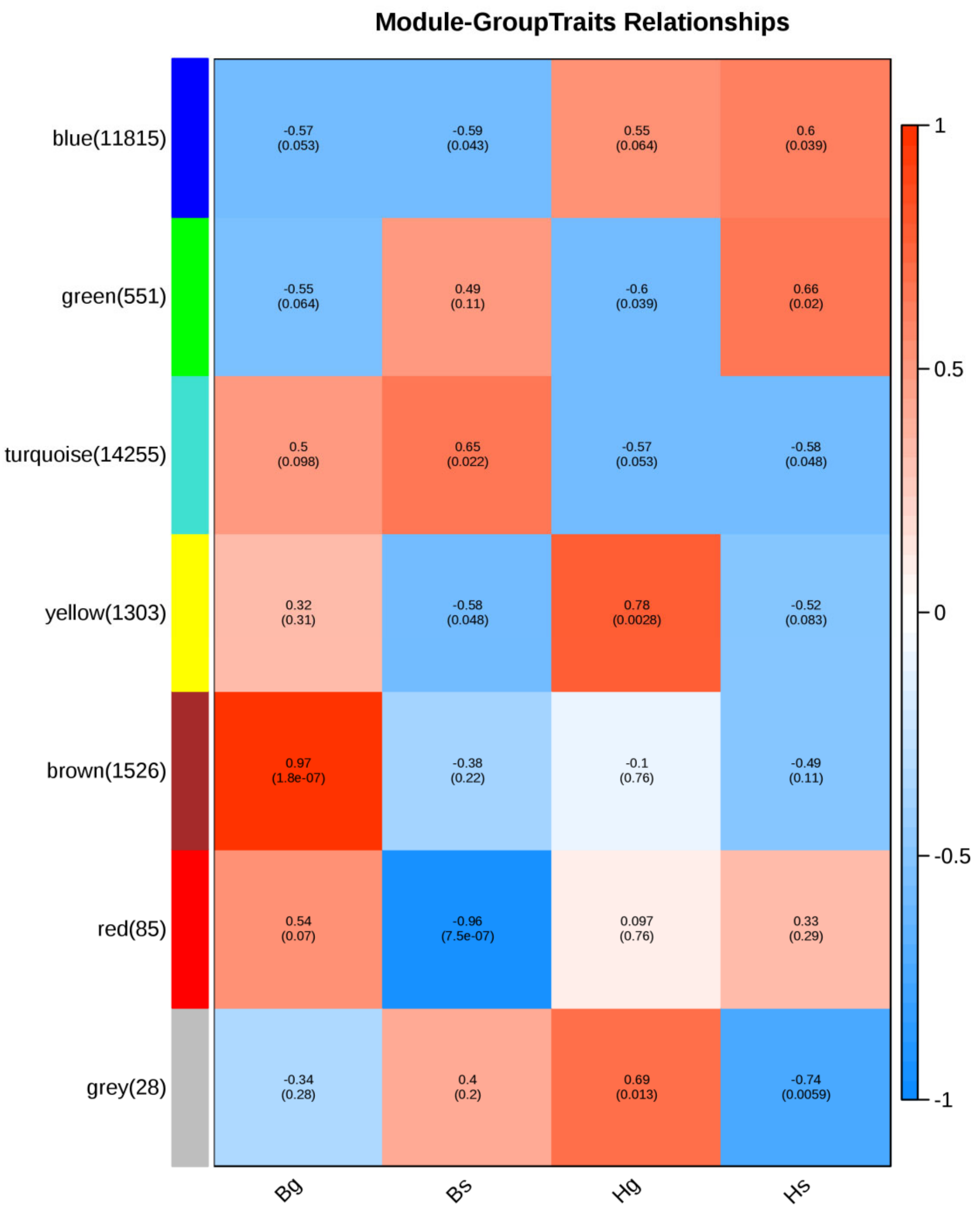


**Figure S6.** Module Group Trait Correlation
